# Comparative effects of different power settings for achieving transmural isolation of the left atrial posterior wall with radiofrequency energy

**DOI:** 10.1093/europace/euae265

**Published:** 2024-10-22

**Authors:** Vincenzo Mirco La Fazia, Nicola Pierucci, Marco Schiavone, Paolo Compagnucci, Sanghamitra Mohanty, Carola Gianni, Domenico Giovanni Della Rocca, Rodney Horton, Amin Al-Ahmad, Luigi Di Biase, Antonio Dello Russo, Carlo Lavalle, Giovanni Forleo, Claudio Tondo, Andrea Natale

**Affiliations:** Texas Cardiac Arrhythmia Institute, St David's Medical Center, 3000 N Interstate Hwy 35 Ste 700, Austin, TX 78705, USA; Department of Cardiovascular, Respiratory, Nephrological, Anesthesiological and Geriatric Sciences, ‘Sapienza’ University of Rome, Rome, Italy; Department of Clinical Electrophysiology and Cardiac Pacing, Centro Cardiologico Monzino, IRCCS, Milan, Italy; Cardiology and Arrhythmology Clinic, University Hospital ‘Ospedali Riuniti’, Ancona, Italy; Texas Cardiac Arrhythmia Institute, St David's Medical Center, 3000 N Interstate Hwy 35 Ste 700, Austin, TX 78705, USA; Texas Cardiac Arrhythmia Institute, St David's Medical Center, 3000 N Interstate Hwy 35 Ste 700, Austin, TX 78705, USA; Texas Cardiac Arrhythmia Institute, St David's Medical Center, 3000 N Interstate Hwy 35 Ste 700, Austin, TX 78705, USA; Heart Rhythm Management Centre, Postgraduate Program in Cardiac Electrophysiology and Pacing, Brussels, Belgium; Texas Cardiac Arrhythmia Institute, St David's Medical Center, 3000 N Interstate Hwy 35 Ste 700, Austin, TX 78705, USA; Texas Cardiac Arrhythmia Institute, St David's Medical Center, 3000 N Interstate Hwy 35 Ste 700, Austin, TX 78705, USA; Texas Cardiac Arrhythmia Institute, St David's Medical Center, 3000 N Interstate Hwy 35 Ste 700, Austin, TX 78705, USA; Department of Electrophysiology, Albert Einstein College of Medicine, Bronx, NY, USA; Cardiology and Arrhythmology Clinic, University Hospital ‘Ospedali Riuniti’, Ancona, Italy; Department of Cardiovascular, Respiratory, Nephrological, Anesthesiological and Geriatric Sciences, ‘Sapienza’ University of Rome, Rome, Italy; Cardiology Unit, Luigi Sacco University Hospital, Milan, Italy; Department of Clinical Electrophysiology and Cardiac Pacing, Centro Cardiologico Monzino, IRCCS, Milan, Italy; Department of Biomedical, Surgical and Dental Sciences, University of Milan, Milan, Italy; Texas Cardiac Arrhythmia Institute, St David's Medical Center, 3000 N Interstate Hwy 35 Ste 700, Austin, TX 78705, USA; Interventional Electrophysiology, Scripps Clinic, San Diego, CA 92093, USA; Metro Health Medical Center, Case Western Reserve University School of Medicine, Cleveland, OH 44109, USA; Department of Biomedicine and Prevention, Division of Cardiology, University of ‘Tor Vergata’, Rome 00133, Italy

**Keywords:** Atrial fibrillation, Posterior wall ablation, Epicardial mapping, Lesion transmurality

## Introduction

Extending the ablation for atrial fibrillation (AF) beyond the pulmonary veins (PVs) into the posterior wall (PW) has the advantage of targeting the greatest amount of arrhythmogenic tissue.^1^ Data on how to achieve durable PW isolation (PWI) are lacking. Previous studies have shown the role of epicardial fibres in arrhythmia recurrence.^2^ The optimal power setting required to achieve transmural PW lesions is unclear. The aim of our study is to report the acute and chronic outcomes of different power strategies (40 W vs. 50 W vs. 90 W).

## Methods

This was a multicentre, prospective registry, enrolling consecutive patients undergoing ablation for persistent AF at four centres: Texas Cardiac Arrhythmia Institute, Austin, USA; Centro Cardiologico Monzino, Milan, Italy; Ospedale Luigi Sacco, Milan, Italy; and Policlinico Umberto I, Roma, Italy.

Patient data were prospectively collected in an institutional review board-approved database.

All patients underwent PWI during the index procedure. During redo procedures, endocardial mapping was performed using LASSO™ or PentaRay™ catheters. Epicardial mapping was performed with Advisor™ HDGrid, PentaRay™, Thermocool Smarttouch™, or QDOT-Micro™ catheters.

Patients were divided into three groups based on the ablation strategy: Group 1 received high-power short duration (HPSD) with 50 W/>10–15 s, Group 2 received HPSD with 40 W/10–15 s, and Group 3 received very high-power short duration (vHPSD) at 90 W/4 s. Catheter was continuously dragged in Groups 1 and 2. A point-by-point strategy was used in Group 3 with an inter-lesion distance of 4 mm. Contact force used was 10–15*g*.

We divided the PW into two anatomical regions: a central part (between the 4PVs) and an inferior part (below the line joining the inferior borders of the inferior PV-encircling lesions to the coronary sinus).

The primary study endpoint was the assessment of chronic transmural PW entrance block achieved during index endocardial PWI, defined as evidence of dense scar [i.e. electrical silence confirmed by the absence of near field electrograms (EGMs)] in both the endocardial and epicardial surfaces of the PW.

Continuous variables are reported as mean ± standard deviation and categorical variables as count and percentage. Fisher’s exact tests were used to compare categorical variables.

## Results

Our study included 12 patients (age 65 ± 7years; 7 males), with persistent AF (mean AF duration: 22 ± 8 months), who underwent AF ablation. In Group 1, four patients were in AF and two patients in sinus rhythm; in Groups 2 and 3, all patients were in AF at the beginning of the procedure.

Epicardial validation of PWI was carried out in redo procedures for seven patients due to chronic effusion at an average of 8 ± 2 months post-procedure, in one case due to VT ablation 4 months after the initial procedure, and in two cases before hybrid convergent redo ablation at an average of 12 ± 6 months (overall time between endocardial PWI and direct epicardial mapping 8 ± 2 months).

In patients with effusion, pericardial access was obtained, and 300 ± 120 cc of serous fluid was drained before starting the ablation with no haemodynamic changes. Steroids were instilled before removing the epicardial sheath at the end of the procedure.

In Group 1 (*n* = 6), five patients received ablation of both central and inferior PW. Subsequent endocardial mapping showed complete electrical silence across the entire PW. However, epicardially, residual signals were detected in the inferior part in one patient. Two patients exhibited AF that organized into AFl and was terminated epicardially on the lower part. In another two patients, AF termination occurred on the left atrial appendage (LAA) side, while cardioversion was performed in the remaining two patients.

**Figure 1:**
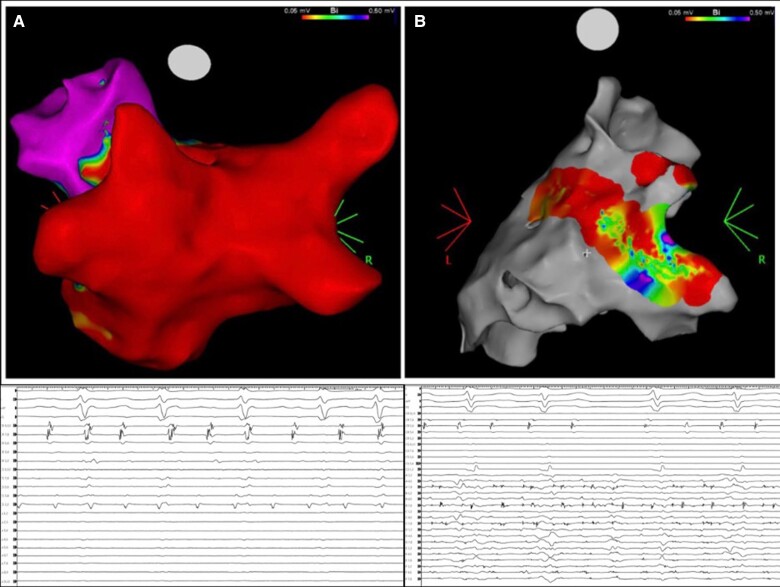
Non-transmural PWI using very high-power short-duration radiofrequency energy. (*A*) Endocardial voltage map and EGMs showing ‘apparent’ PWI. (*B*) Epicardial voltage map and EGMs showing remaining activity on PW.

In Group 2 (*n* = 3), one patient underwent ablation only of the central PW, while the remaining two underwent ablation of both central and inferior PW. Endocardial silence was detected in the ablated regions during mapping. However, epicardial mapping indicated residual signals across the entire PW. In this group, one patient AF termination occurred during epicardial ablation of the central PW (*Figure 1*). For the remaining two patients, pre-procedural electrical cardioversion was performed to restore SR.

In Group 3 (*n* = 3), patients underwent ablation only of the central PW. Endocardial mapping confirmed silence in the targeted region. Epicardial mapping revealed residual signals across all PW areas. In this group, one patient achieved AF termination during epicardial ablation on the LAA side, while the remaining two had AF organization into atrial flutter and termination at the inferior PW region.

When comparing the three groups, epicardial isolation of all PW was observed in five patients (83.3%) in Group 1, whereas no patients (0%) in Groups 2 and 3 exhibited epicardial isolation (*P* = 0.001).

## Discussion

To the best of our knowledge, this is the first study to compare different power settings in relation to PWI to assess lesion transmurality.

Non-transmural PWI may contribute to arrhythmia recurrence by creating a substrate for endo-epicardial dissociation and transmural re-entry, potentially mediated by the septopulmonary bundle.^3–5^ This was observed only with vHPSD and HPSD with 40 W/10–15 s. Our findings are in accordance with previous studies that showed the deepest penetration with 50 W compared with 40 and 90 W strategy.^6,7^

Another important finding is the role of the inferior part of PW in arrhythmogenicity. Indeed, epicardial ablations resulted in AF conversion into AFl, which could be successfully terminated in the inferior part of the PW. The significance of the inferior part of is underscored by the higher long-term success rates achieved with the convergent procedure. Indeed, this technique affords the surgeon improved access to the inferior PW region (often neglected during endocardial ablation), thereby enhancing the effectiveness of PWI.^8^ Pulsed-field ablation has recently been shown to achieve PW transmural lesions due to its capability to easily deliver larger lesions extendable to the inferior PW.^9^

The generalizability of this study is limited by the relatively small sample size. We cannot exclude that fluid drainage impacted arrhythmia termination. However, following drainage, there was no haemodynamic change, and AF was organized into AFl in most of the patients.

## Conclusion

Results from this small consecutive series suggest that HPSD with 50 W/10–15 s was associated with transmural lesion on central PW compared with other power settings evaluated.

Furthermore, the role of the inferior PW in the arrhythmogenicity of the left atrium is highlighted, suggesting that targeted ablation in this area can increase procedural success.

## Data Availability

Data are available on reasonable request. The data that support the findings of this study are available from the corresponding author upon reasonable request.
